# Integrative Analyses of Genes Associated with Fulminant Type 1 Diabetes

**DOI:** 10.1155/2020/1025857

**Published:** 2020-10-06

**Authors:** Xiaofeng Ye, Tianshu Zeng, Wen Kong, Lu-lu Chen

**Affiliations:** ^1^Department of Endocrinology, Union Hospital, Tongji Medical College, Huazhong University of Science and Technology, Wuhan 430022, China; ^2^Hubei Provincial Clinical Research Center for Diabetes and Metabolic Disorders, Wuhan 430022, China

## Abstract

**Objective:**

Fulminant type 1 diabetes (FT1D) is a type of type 1 diabetes, which is characterized by rapid onset of disease and severe metabolic disorders. We intend to screen for crucial genes and potential molecular mechanisms in FT1D in this study.

**Method:**

We downloaded GSE44314, which includes six healthy controls and five patients with FT1D, from the GEO database. Identification of differentially expressed genes (DEGs) was performed by NetworkAnalyst. The Gene Ontology (GO) and Kyoto Encyclopedia of Genes and Genomes (KEGG) enrichment analyses of DEGs were screened by an online tool—Database for Annotation, Visualization, and Integration Discovery (DAVID). Protein-protein interaction (PPI) network and hub genes among DEGs were analyzed by NetworkAnalyst. And we also use NetworkAnalyst to find out the microRNAs (miRNAs) and transcription factors (TFs) which regulate the expression of DEGs.

**Result:**

We identified 130 DEGs (60 upregulated and 70 downregulated DEGs) between healthy controls and FT1D patients. GO analysis results revealed that DEGs were mostly enriched in generation of precursor metabolites and energy, neurohypophyseal hormone activity, and mitochondrial inner membrane. KEGG pathway analysis demonstrated that DEGs were mostly involved in nonalcoholic fatty liver disease. Results indicated that NCOA1, SRF, ERBB3, EST1, TOP1, UBE2S, INO80, COX7C, ITGAV, and COX6C were the top hub genes in the PPI network. Furthermore, we recognized that LDLR, POTEM, IFNAR2, BAZ2A, and SRF were the top hub genes in the miRNA-target gene network, and SRF, TSPAN4, CD59, ETS1, and SLC25A25 were the top hub genes in the TF-target gene network.

**Conclusion:**

Our study pinpoints key genes and pathways associated with FT1D by a sequence of bioinformatics analysis on DEGs. These identified genes and pathways provide more detailed molecular mechanisms of FT1D and may provide novel therapeutic targets.

## 1. Introduction

Fulminant type 1 diabetes (FT1D) is a novel type of type 1 diabetes (T1DM) raised by Imagawa et al. in 2000 [1], which is featured by abrupt disease onset, no C-peptide secretion, negative islet-related autoantibodies, and elevated pancreatic enzymes. At first, FT1D was identified as idiopathic T1DM because patients with FT1D lack autoimmune markers such as protein tyrosine phosphatase antibody or glutamic acid decarboxylase autoantibody. Over the past 20 years, the understanding of FT1D has increased. And a sequence of studies indicated that the immunity has a role in the occurrence and development of FT1D, which convinced that FT1D is possibly an autoimmune disease [2–4].

There are studies that reported that genetic and environmental factors take part in the initiation and progression of FT1D. Numbers of studies indicated that CTLA-4, HLA-B, and HLA DR-DQ are related with FT1D [5–7]. Many studies advocate that in FT1D, immune response against viral infection in islets caused the *β* cell destruction [8–10]. Numerous virus infections were covered in FT1D patients, including coxsackievirus, enterovirus, and human cytomegalovirus [11–13]. Genes such as lymphocyte cytosolic protein 1, melanoma differentiation-associated protein 5, DEAD box helicase 5, and C-X-C motif chemokine 10, which take part in the virus infection, have been proved to be associated with the pathogenesis of FT1D [3, 11, 14]. To further reveal the mechanism of FT1D, a microarray data numbered GSE44314 was deposited by Nakata et al., and it has reported that NKG2E-CD94 were significantly reduced in FT1D, indicating that the reduced expression of NK activating receptor gene and low proportion of NK cells are probably involved in the progression of FT1D [15]. However, there are no studies that had reported the possible regulatory mechanisms of transcription factors (TFs) and microRNAs (miRNAs) related to the development of FT1D.

In our study, we reanalyzed the dataset of GSE44314 by the method of bioinformatics, which includes screening differentially expressed genes (DEGs), functional enrichment analysis, protein-protein interaction (PPI) analysis, and the regulatory TFs/miRNAs related to DEG prediction. Through these analyses, we expect to determine novel insights for the knowledge of FT1D and provide more detailed molecular mechanisms underlying the development of FT1D.

## 2. Materials and Methods

### 2.1. Microarray Data

We downloaded the gene expression profile data of GSE44314 from the Gene Expression Omnibus (GEO) database in the National Center for Biotechnology Information (NCBI, https://www.ncbi.nlm.nih.gov/geo/). The microarray data was based on the platform of GPL6480 (Agilent-014850 Whole Human Genome Microarray 4x44K G4112F). The datasets available in this analysis were uploaded by Nakata et al. [15], which include 11 samples, containing 6 healthy controls and 5 patients with FT1D.

### 2.2. Identification of Differentially Expressed Genes

NetworkAnalyst [16, 17] (https://www.networkanalyst.ca), a website for integrative statistical and visualizing tool, was used to determine the DEGs between healthy controls and FT1D patients. The cutoff of the *P* value was adjusted to 0.05, and ∣log fold change |  (∣log FC∣) > 0.585 for the DEG discrimination, using the false discovery rate (FDR) found on the Benjamini-Hochberg program and moderated *t*-test based on the Limma algorithm.

### 2.3. Functional and Pathway Enrichment Analysis

We used an online tool named DAVID [18] (https://david.ncifcrf.gov/) in conducting the Gene Ontology (GO) term [19] and Kyoto Encyclopedia of Genes and Genomes (KEGG) [20] pathway enrichment analyses of DEGs, with the thresholds of count ≥ 2 and *P* value < 0.05.

### 2.4. Protein-Protein Interaction (PPI) Network Analysis and Hub Gene Searching

Based on the analyzed DEGs, NetworkAnalyst [21] was used to perform the PPI Network identification with a hypergeometric algorithm, and *P* < 0.05 was identified as having statistically significant differences. Besides, we used NetworkAnalyst to recognize the most significant modules of hub genes using the “module explorer tool,” found on the random walk-dependent Walktrap algorithm.

### 2.5. Prediction of Target Gene-MicroRNA Network

The gene expression was affected by microRNAs in a disease condition through posttranscriptional control. In the present study, the online tool NetworkAnalyst [17] was used to search the miRNAs associated with DEGs, which integrates microRNA databases miRTarBase (http://mirtarbase.mbc.nctu.edu.tw/php/download.php) [22] and TarBase (http://diana.imis.athena-innovation.gr/DianaTools/index.php?r=tarbase/index) [23].

### 2.6. Prediction of Target Gene-Transcription Factor Network

The gene expression was influenced by TFs in a disease condition by transcriptional control. In our study, NetworkAnalyst [17] was used for recognizing the TFs associated with DEGs, which combines TF database JASPAR (http://jaspar.genereg.net/) [24].

## 3. Results

### 3.1. Identification of Differentially Expressed Genes in Fulminant Type 1 Diabetes

**Figure 1:**
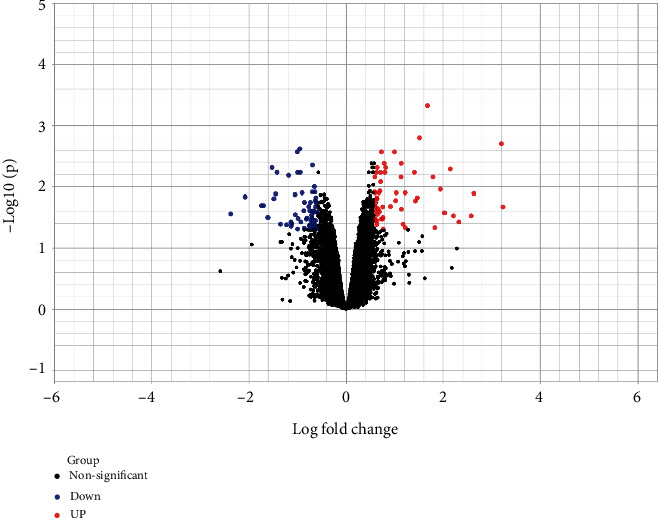
Volcano plot of differentially expressed genes. Genes with a significant change of more than 1.5-fold were selected.

**Figure 2:**
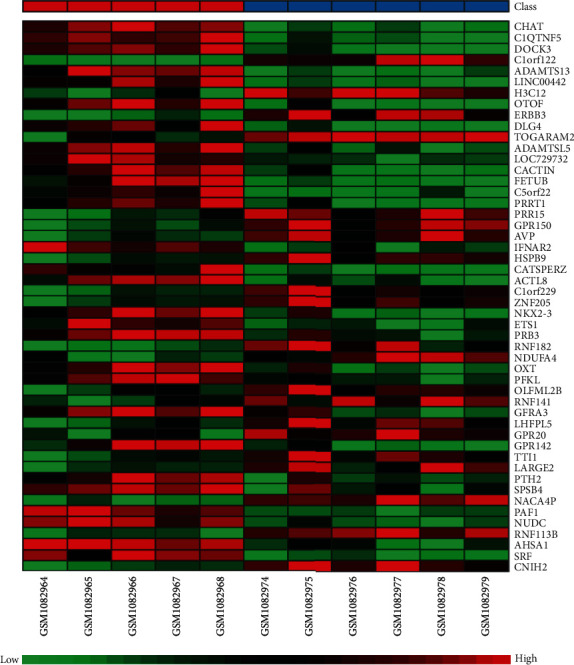
Heat map of differentially expressed genes. The abscissa represents different samples, and the ordinate represents different genes. The red boxes indicate upregulated genes, and the green boxes indicate downregulated genes.

We identified 130 DEGs in FT1D patients compared to healthy controls in total, including 60 upregulated genes and 70 downregulated genes (Supplementary Table 1). We draw a volcano plot of the DEGs (Figure 1) and a hierarchical clustering heat map of DEGs (Figure 2). It turned out that these DEGs were well distinguished between the FT1D group and the healthy control group. NK2 homeobox 3 (NKX2-3) and Ring finger protein 182 (RNF182) were, respectively, identified as the most significantly upregulated and downregulated genes in FT1D patients.

### 3.2. Functional Enrichment Analysis

We recognized 21 Gene Ontology terms (Table 1) and 5 KEGG pathways (Table 2) when analyzed with DAVID. The DEGs were mainly focused on the generation of precursor metabolites and energy, hydrogen ion transmembrane transport, and mitochondrial electron transport, cytochrome c to oxygen by biological process (BP) analysis. For the cellular component (CC) group, mitochondrial inner membrane, extracellular space, and cell junction were the enriched terms. Molecular function (MF) analysis showed that the DEGs were remarkably focused on neurohypophyseal hormone activity, cytochrome c oxidase activity, and neuregulin binding. Moreover, the KEGG pathway analysis indicated that the DEGs were significantly involved in nonalcoholic fatty liver disease, Huntington's disease, Alzheimer's disease, and Parkinson's disease as well as oxidative phosphorylation.

### 3.3. PPI Network and Hub Gene Identification

**Figure 3:**
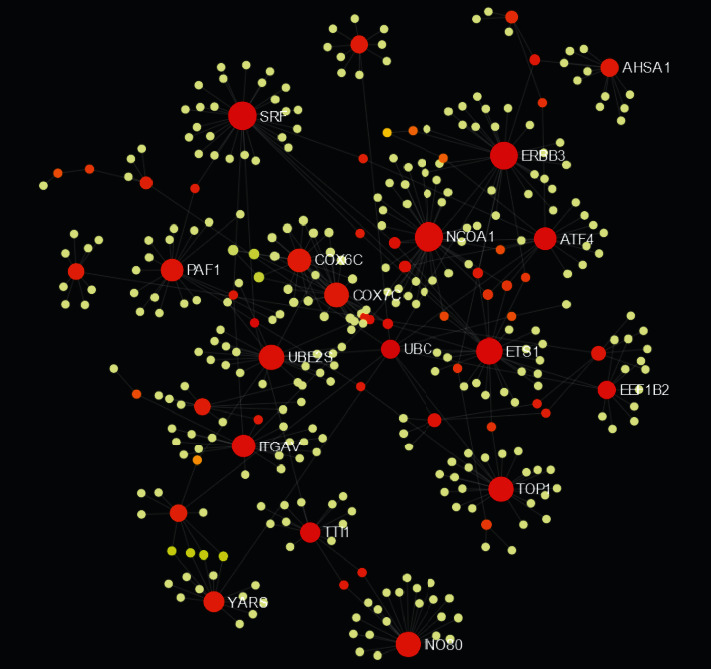
Protein-protein interaction network of the differentially expressed genes. Red and orange nodes stand for hub genes.

There were 363 nodes and 409 edges in the PPI network (Figure 3). In this PPI network, sixteen genes with degrees > 10 were found as key genes (Table 3). The node size is influenced by the fold change between FT1D patients and healthy controls, and the red or orange color nodes indicate that they have a higher score. The core of the whole PPI network was the most key genes in this cluster, including NCOA1, SRF, ERBB3, ETS1, TOP1, UBE2S, INO80, COX7C, ITGAV, COX6C, ATF4, PAF1, YARS, TTI1, UBC, EEF1B2, and AHSA1. Thence, the seventeen genes were recognized as the hub genes.

### 3.4. miRNA-DEG and TF-DEG Regulating Network Analysis

**Figure 4:**
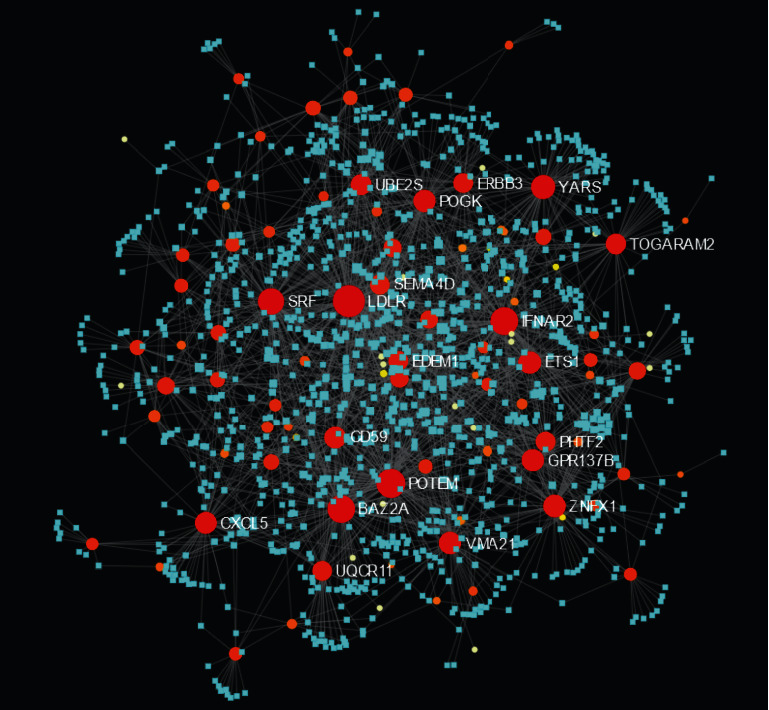
Target gene-miRNA regulatory network. Red and orange nodes stand for differentially expressed genes; blue diamonds stand for miRNA.

**Figure 5:**
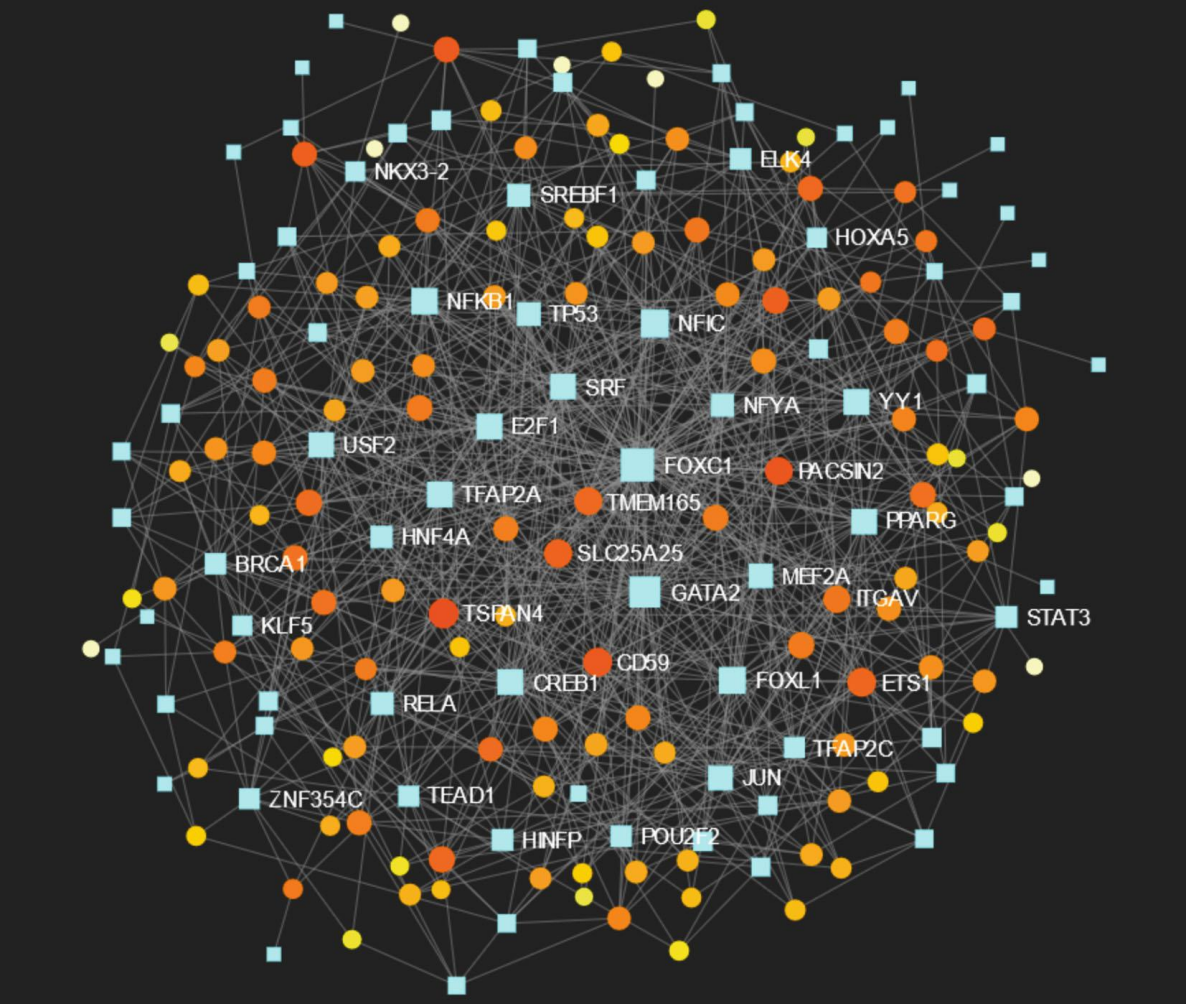
Transcription factor-target DEG regulatory network. Orange and yellow nodes stand for differentially expressed genes; blue diamonds stand for transcription factors.

The miRNAs and TFs for DEGs are displayed in Figures 4 and 5, respectively. The top five targeted genes regulated by miRNA are shown in Supplementary Table 2. It turned out that 167 miRNAs regulate LDLR, 124 miRNAs regulate POTEM, 109 miRNAs regulate IFNAR2, 107 miRNAs regulate BAZ2A, and 92 miRNAs regulate SRF. The top five targeted genes regulated by TFs are shown in Supplementary Table 3. It turned out that 25 TFs regulate SRF, 18 TFs regulate TSPAN4, 16 TFs regulate CD59, 16 TFs regulate ETS1, and 15 TFs regulate SLC25A25.

## 4. Discussion

FT1D is a disease with a state of insulin dependency due to the rapid destruction of almost all pancreatic *β* cells, which causes the radical onset of ketoacidosis in a few days after the appearance of hyperglycemic symptoms [25–27]. It has been reported that most of the patients with FT1D are found in East Asia, but recently, Western countries also reported this disease [8, 28, 29]. FT1D makes up about 20% of abrupt-onset T1DM cases in Japan [8]. It is important to understand the molecular mechanisms of FT1D. We downloaded and analyzed a dataset (GSE44314) that contains five FT1D patients and six healthy controls from the GEO database. We identified 130 DEGs in total, including 60 upregulated DEGs and 70 downregulated DEGs. Among the 130 DEGs, we noticed that programmed cell death-1 (PD-1) was downregulated in FT1D patients. PD-1 is a critical member of the B7-CD28 family and is one of the important costimulatory molecules [30]. PD-1 can regulate the T cell response and keep maintaining peripheral tolerance by delivering critical inhibitory signals [30]. Inhibiting the PD-1 pathway would bring about excessive T cell proliferation, failure of tolerance, and autoimmune activation [31]. Therefore, PD-1 has gained popularity in the treatment of several advanced cancers [32, 33]. Studies have proved that treatment with PD-1 inhibitors can cause FT1D [34–36]. And the termination of anti-PD1 antibody therapy may preserve inherent insulin secretion capacity in “anti-PD1 antibody-induced” FT1D [37]. It seems that PD-1 should be upregulated in FT1D, which is totally opposite to our result. Various researchers have identified that cellular immunity, especially T cell, played a crucial role in *β* cell destruction in FT1D [38–40]. However, a Japanese study that compares PD-1 expression in peripheral CD4+ T cells between type 1A diabetes (classical type 1 diabetes), FT1D, and healthy controls found that there is no difference between FT1D and healthy controls in PD-1 expression and that there is lower PD-1 expression in CD4+ T cells in patients with type 1A diabetes [41]. Different studies have different conclusions in PD-1 expression in FT1D, which need further studies to confer this question and explore how PD-1 take part in the occurrence and progression of FT1D. Among the increased DEGs, NK2 homeobox 3 (NKX2-3) is the most upregulated gene in FT1D, and an animal study has indicated that NKX2-3 is related to T1DM [42], but further study is needed to figure out how NKX2-3 acts in FT1D.

In the current study, the most significant GO BP term for DEGs is generation of precursor metabolites. UQCR11, COX7C, and COX6C are the new biomarkers for the progression of FT1D. The most significant GO MF term for DEGs is neurohypophyseal hormone activity. Arginine vasopressin (AVP) and oxytocin are associated with type 2 diabetes but are new biomarkers for the progression of FT1D. The most significant GO CC term for DEGs is mitochondrial inner membrane. NDUFA4, SLC25A25, ROMO1, MRPL30, and NDUFB1 are novel biomarkers for the development of FT1D. Nonalcoholic fatty liver disease is the most significant KEGG pathway for DEGs. Activation of activating transcription factor 4 (ATF4) contributes to diabetic hepatotoxicity by ER stress [43]. Besides, ATF4 is a transcription factor implicated in *β* cell survival and susceptibility to stress [44]. ATF4 is a new biomarker for the progression of FT1D. Parkinson's disease, Alzheimer's disease, and Huntington's disease also are significant KEGG pathways for DEGs. Diabetes mellitus (DM) adversely affects multiple organ systems, including the brain [45]. These evidences suggest that FT1D may also lead to neurodegenerative diseases and adversely affect cognition. Discs large MAGUK scaffold protein 4 (DLG4) is related to neurological disorders and type 2 diabetes [46–48]; DLG4 is a new biomarker for the progression of FT1D.

In the present study, NCOA1, SRF, ERBB3, ETS1, TOP1, UBE2S, INO80, COX7C, ITGAV, and COX6C were recognized as top 10 hub genes in the PPI network. A genome-wide meta-analysis study confirmed that nuclear receptor coactivator 1 (NCOA1) is a T1DM susceptibility gene [49]. An animal study suggests that serum response factor (SRF) is decreased in diabetic nephropathy compared to healthy controls [50]. Many studies confirmed that ERBB3 was the most important T1DM association locus in the non-HLA gene [51–53]. ETS proto-oncogene 1 (EST1) was found associated with T1DM in the NOD mouse and then confirmed in human population [54–56]. Tissues derived from the T1DM animals show that DNA topoisomerase I (TOP1) activity and enzyme protein level decreased, whereas the enzyme mRNA level was not altered, which demonstrates that TOP1 activity is regulated by high glucose levels and may lead to the pathogenesis of diabetic complications [57]. Ubiquitin-conjugating enzyme E2 (UBE2S) takes part in T1DM by enhancing M2 macrophage polarization [58]. Jin et al. compared integrin subunit alpha V (ITGAV) expression between diabetic nephropathy and normal human kidney and found that ITGAV is higher in diabetic nephropathy [59]. Although there are evidences that the hub genes are contacted with T1DM, they are novel biomarkers for the development of FT1D.

LDLR, POTEM, IFNAR2, BAZ2A, and SRF were identified as top five targeted genes in the miRNA-target gene regulatory network. Low-density lipoprotein receptor (LDLR) is increased in a NOD mouse compared with a nondiabetic mouse [60]. A study in Ins2(Akita)Ldlr^−^/^−^ mice revealed that lack of LDLR will accelerate atherosclerosis in T1DM animals [61]. When lacking the r type II interferon receptor (IFNAR2), diabetes happened only in female NOD mice [62]. POTEM and BAZ2A are novel biomarkers for the development of FT1D. SRF, TSPAN4, CD59, ETS1, and SLC25A25 were identified as top five targeted genes in the TF-DEG regulatory network. Due to the lack of complement regulatory protein CD59, the development of diabetes-induced atherosclerosis in mice is accelerated [63]. Besides, CD59 is reduced in diabetic subjects compared with healthy controls [64]. Tetraspanin 4 (TSPAN4) is a new biomarker for the progression of FT1D.

We noticed that there are two bioinformatics analysis of type 1 diabetes, and there are some the same conclusions between our study and theirs [65, 66]. Fang et al. reported that programmed cell death ligand 1 (PD-L1) was upregulated in the new-onset T1DM samples [66]. This is identical with our result. PD-1/PD-L1 is a negative modulatory signaling pathway for activation of T cell. The upregulated PD-L1 and downregulated PD-l cause the same result, which are the inactivation of T cell and the progression of immune tolerance, which play a protective role in the pathogenesis of T1DM. Liu et al. found that HLA-DQA1 and HLA-DRB4 might be targets for the treatment of T1D, and IL8 is likely to be a new marker for the diagnosis of T1D [65]. These results indicated that T1DM is an autoimmune disease, which is in accordance with our result.

## 5. Conclusions

Our data provide a comprehensive bioinformatics analysis of DEGs to search molecular mechanisms related to the progression of FT1D. We found a set of useful genes for future research into the molecular mechanisms of FT1D progression, while further molecular biological experiments are needed to confirm the effect of these DEGs in the progression of FT1D.

## Figures and Tables

**Table 1:** The results of Gene Ontology (GO) of DEGs ranked by *P* value.

Term	Count	*P* value	Genes
GO-BPs			
Generation of precursor metabolites and energy	4	0.004	AVP, UQCR11, COX7C, COX6C
Hydrogen ion transmembrane transport	4	0.006	NDUFA4, UQCR11, COX7C, COX6C
Mitochondrial electron transport, cytochrome c to oxygen	3	0.006	NDUFA4, COX7C, COX6C
Extrinsic apoptotic signaling pathway in the absence of ligand	3	0.017	MOAP1, ERBB3, ITGAV
Positive regulation of female receptivity	2	0.018	NCOA1, OXT
Positive regulation of gene expression	6	0.02	AMH, ATF4, AVP, LDLR, ERBB3, GPER1
Maternal aggressive behavior	2	0.024	AVP, OXT
Hyperosmotic salinity response	2	0.029	AVP, OXT
Cellular response to lipopolysaccharide	4	0.03	TNFRSF1B, ADAMTS13, PAF1, CACTIN
Social behavior	3	0.033	AVP, OXT, DLG4
Positive regulation of apoptotic process	6	0.034	MOAP1, ATF4, NCOA1, ARHGEF6, GPER1, PDCD1
Male mating behavior	2	0.035	NCOA1, OXT
Positive regulation of uterine smooth muscle contraction	2	0.041	OXT, GPER1
Drinking behavior	2	0.041	HTR1B, OXT
Positive regulation of cytosolic calcium ion concentration	4	0.046	AVP, OXT, DLG4, GPER1
GO-MFs			
Neurohypophyseal hormone activity	2	0.011	AVP, OXT
Cytochrome c oxidase activity	3	0.013	NDUFA4, COX7C, COX6C
Neuregulin binding	2	0.028	ERBB3, ITGAV
GO-CCs			
Mitochondrial inner membrane	8	0.014	NDUFA4, UQCR11, SLC25A25, COX7C, ROMO1, MRPL30, NDUFB1, COX6C
Extracellular space	14	0.046	INA, AVP, CXCL5, ERBB3, ADAMTS13, OXT, FETUB, AMH, IFNAR2, C1QTNF5, CLEC3B, CD59, SEMA4D, PRSS33
Cell junction	7	0.05	CNIH2, OTOF, PRRT1, DLG4, PAF1, GPER1, GPR142

**Table 2:** The results of Kyoto Encyclopedia of Genes and Genomes (KEGG) of DEGs ranked by *P* value.

Term	Count	*P* value	Genes
Nonalcoholic fatty liver disease (NAFLD)	6	0.0017	NDUFA4, ATF4, UQCR11, COX7C, NDUFB1, COX6C
Huntington's disease	6	0.0048	NDUFA4, UQCR11, DLG4, COX7C, NDUFB1, COX6C
Oxidative phosphorylation	5	0.0071	NDUFA4, UQCR11, COX7C, NDUFB1, COX6C
Parkinson's disease	5	0.0089	NDUFA4, UQCR11, COX7C, NDUFB1, COX6C
Alzheimer's disease	5	0.0158	NDUFA4, UQCR11, COX7C, NDUFB1, COX6C

**Table 3:** Sixteen genes with degrees < 10 in the protein-protein interaction network of the differentially expressed genes.

Gene	Regulation	Degree	Betweenness	Expression
ETS1	Up	26	15103.54	1.145
AHSA1	Up	11	3565	0.82
TOP1	Up	23	10312.37	0.764
NCOA1	Up	34	9967.16	0.752
PAF1	Up	18	5908.12	0.732
SRF	Up	31	22498.06	0.647
YARS	Up	15	4237.33	0.644
INO80	Up	22	7030.5	0.606
ITGAV	Down	20	9973.24	-0.603
ATF4	Down	18	11878.52	-0.705
COX6C	Down	20	3314.17	-0.759
COX7C	Down	22	4037.83	-0.801
EEF1B2	Down	11	11109	-0.817
UBE2S	Down	23	7532.83	-0.858
TTI1	Down	14	11460.5	-1.226
ERBB3	Down	29	19037.55	-1.422

## Data Availability

The data used to support the findings of this study are included within the article.

## Supplementary Materials

Supplementary Materials Supplementary Table 1: the list of all differentially expressed genes. Supplementary Table 2: the top five targeted genes regulated by miRNA. Supplementary Table 3: the top five targeted genes regulated by transcription factor.
