# Supplementary material for: Integrative Analyses of Genes Associated with Fulminant Type 1 Diabetes
Source: J Immunol Res. 2020 Oct 6;2020:1025857. doi: 10.1155/2020/1025857 (PMC7559223; doi:10.1155/2020/1025857)
Supplement: Supplementary Materials — Supplementary Table 1: the list of all differentially expressed genes. Supplementary Table 2: the top five targeted genes regulated by miRNA. Supplementary Table 3: the top five targeted genes regulated by transcription factor. [file 1025857.f1.docx]

Supplementary Table 1 The list of all differentially expressed genes

| Gene | logFC | AveExpr | t | P.Value | adj.P.Val | B |
| --- | --- | --- | --- | --- | --- | --- |
| CHAT | 1.6847 | 6.3142 | 12.245 | 4.39E-08 | 0.000475 | 8.4536 |
| C1QTNF5 | 1.5187 | 6.9892 | 10.279 | 2.96E-07 | 0.001601 | 6.9188 |
| DOCK3 | 3.1998 | 8.454 | 9.6895 | 5.56E-07 | 0.002005 | 6.3866 |
| C1orf122 | -1.0044 | 9.9523 | -8.7913 | 1.55E-06 | 0.002729 | 5.5006 |
| ADAMTS13 | 1.0035 | 7.0754 | 8.6789 | 1.77E-06 | 0.002729 | 5.383 |
| LINC00442 | 1.1441 | 5.9351 | 8.0067 | 4.04E-06 | 0.0042 | 4.6453 |
| H3C12 | -1.5261 | 10.28 | -7.5059 | 7.72E-06 | 0.004909 | 4.0561 |
| OTOF | 2.1465 | 11.27 | 7.4207 | 8.64E-06 | 0.005192 | 3.9524 |
| ERBB3 | -1.4223 | 6.9402 | -7.2615 | 1.07E-05 | 0.005874 | 3.7556 |
| DLG4 | 1.4133 | 7.9205 | 7.095 | 1.34E-05 | 0.005874 | 3.546 |
| TOGARAM2 | -1.1813 | 6.952 | -6.8754 | 1.82E-05 | 0.006568 | 3.2631 |
| ADAMTSL5 | 1.7959 | 8.1304 | 6.7825 | 2.08E-05 | 0.006995 | 3.1411 |
| LOC729732 | 1.1395 | 9.6826 | 6.7764 | 2.09E-05 | 0.006995 | 3.133 |
| CACTIN | 1.9508 | 10.954 | 6.3486 | 3.89E-05 | 0.011056 | 2.5541 |
| FETUB | 1.2224 | 5.7654 | 6.0664 | 5.92E-05 | 0.01266 | 2.1564 |
| C5orf22 | 1.0378 | 6.946 | 6.0606 | 5.97E-05 | 0.01266 | 2.1481 |
| PRRT1 | 2.6319 | 8.7325 | 6.0334 | 6.22E-05 | 0.012937 | 2.1092 |
| PRR15 | -1.45 | 8.0207 | -6.0113 | 6.43E-05 | 0.013127 | 2.0773 |
| GPR150 | -1.0512 | 16.07 | -5.967 | 6.88E-05 | 0.013531 | 2.0134 |
| AVP | -2.0709 | 8.3433 | -5.8764 | 7.91E-05 | 0.014742 | 1.8815 |
| IFNAR2 | 1.4748 | 7.7162 | 5.8361 | 8.41E-05 | 0.015163 | 1.8225 |
| HSPB9 | -1.4871 | 9.3562 | -5.7422 | 9.73E-05 | 0.015779 | 1.6839 |
| CATSPERZ | 1.0253 | 6.5393 | 5.6333 | 0.000115 | 0.016952 | 1.5215 |
| ACTL8 | 1.4346 | 6.2259 | 5.6027 | 0.000121 | 0.017143 | 1.4755 |
| C1orf229 | -1.6916 | 13.092 | -5.2754 | 0.000204 | 0.020409 | 0.97432 |
| ZNF205 | -1.7329 | 10.377 | -5.2611 | 0.000209 | 0.020409 | 0.95213 |
| NKX2-3 | 3.2318 | 9.315 | 5.1307 | 0.000259 | 0.021358 | 0.74745 |
| ETS1 | 1.1452 | 9.0226 | 5.0335 | 0.000304 | 0.023294 | 0.5932 |
| PRB3 | 2.0311 | 9.6138 | 4.8207 | 0.000433 | 0.02678 | 0.25059 |
| RNF182 | -2.3684 | 6.359 | -4.7796 | 0.000464 | 0.027905 | 0.18371 |
| NDUFA4 | -1.0434 | 9.9519 | -4.7019 | 0.00053 | 0.028796 | 0.056499 |
| OXT | 2.5769 | 10.719 | 4.6436 | 0.000585 | 0.029981 | -0.03953 |
| PFKL | 2.2088 | 10.032 | 4.6411 | 0.000588 | 0.029981 | -0.04356 |
| OLFML2B | -1.607 | 10.126 | -4.5526 | 0.000684 | 0.031888 | -0.19026 |
| RNF141 | -1.1348 | 9.963 | -4.3023 | 0.001057 | 0.037852 | -0.61053 |
| GFRA3 | 2.3225 | 9.4759 | 4.2981 | 0.001064 | 0.037852 | -0.6177 |
| LHFPL5 | -1.1152 | 7.7213 | -4.2522 | 0.001154 | 0.040247 | -0.69564 |
| GPR20 | -1.3505 | 7.2554 | -4.232 | 0.001195 | 0.041048 | -0.73 |
| GPR142 | 1.1796 | 6.7793 | 4.2216 | 0.001218 | 0.041048 | -0.74771 |
| TTI1 | -1.2257 | 9.0298 | -4.1795 | 0.001311 | 0.042083 | -0.81958 |
| LARGE2 | -1.1307 | 8.6257 | -4.1128 | 0.001476 | 0.044425 | -0.93376 |
| PTH2 | 1.2254 | 7.6034 | 4.0415 | 0.001675 | 0.046779 | -1.0565 |
| SPSB4 | 1.8365 | 13.981 | 4.0354 | 0.001694 | 0.046839 | -1.0669 |
| NACA4P | -0.9523 | 10.347 | -9.2597 | 8.98E-07 | 0.002429 | 5.9744 |
| PAF1 | 0.73235 | 9.6703 | 8.838 | 1.46E-06 | 0.002729 | 5.5491 |
| NUDC | 0.78889 | 9.663 | 8.0421 | 3.86E-06 | 0.0042 | 4.6856 |
| RNF113B | -0.68969 | 6.4044 | -7.8471 | 4.95E-06 | 0.004457 | 4.4613 |
| AHSA1 | 0.81987 | 9.4798 | 7.6471 | 6.41E-06 | 0.004909 | 4.2259 |
| SRF | 0.64674 | 6.6961 | 7.5412 | 7.36E-06 | 0.004909 | 4.0988 |
| CNIH2 | -0.99778 | 7.3317 | -7.2123 | 1.14E-05 | 0.005874 | 3.6941 |
| PI4KB | 0.62902 | 9.5656 | 7.159 | 1.23E-05 | 0.005874 | 3.627 |
| CRACR2B | -0.94222 | 8.9175 | -7.0907 | 1.35E-05 | 0.005874 | 3.5405 |
| ACAP1 | 0.80404 | 9.0245 | 7.0102 | 1.51E-05 | 0.005874 | 3.4376 |
| EDEM1 | 0.71497 | 7.7278 | 7.0022 | 1.53E-05 | 0.005874 | 3.4273 |
| POGK | 0.59641 | 7.5067 | 6.7631 | 2.13E-05 | 0.006995 | 3.1155 |
| SEMA4D | 0.71752 | 7.7676 | 6.6176 | 2.63E-05 | 0.008356 | 2.9215 |
| LRRC57 | -0.65145 | 7.0753 | -6.4327 | 3.43E-05 | 0.010036 | 2.6702 |
| BAZ2A | 0.70317 | 8.0142 | 6.2768 | 4.32E-05 | 0.011774 | 2.4541 |
| MRPL30 | -0.69137 | 9.4184 | -6.1901 | 4.91E-05 | 0.012302 | 2.3323 |
| TRARG1 | 0.58971 | 6.0084 | 6.1846 | 4.96E-05 | 0.012302 | 2.3245 |
| NUDT16P1 | -0.63883 | 5.736 | -6.1784 | 5.00E-05 | 0.012302 | 2.3157 |
| MOAP1 | 0.68298 | 7.7755 | 6.1226 | 5.44E-05 | 0.01266 | 2.2366 |
| INO80 | 0.60585 | 6.351 | 6.0998 | 5.63E-05 | 0.01266 | 2.2041 |
| FTHL17 | -0.90429 | 11.931 | -6.0717 | 5.87E-05 | 0.01266 | 2.164 |
| SLC25A25 | -0.61853 | 9.107 | -5.8395 | 8.37E-05 | 0.015163 | 1.8275 |
| GARS1 | 0.64029 | 7.2095 | 5.8215 | 8.60E-05 | 0.015222 | 1.8011 |
| YARS1 | 0.64397 | 7.1178 | 5.7392 | 9.78E-05 | 0.015779 | 1.6795 |
| SPEM2 | -0.6353 | 6.8002 | -5.6424 | 0.000114 | 0.016952 | 1.5351 |
| FMC1 | -0.61288 | 8.3105 | -5.5233 | 0.000137 | 0.017763 | 1.3555 |
| MCEE | -0.61381 | 8.094 | -5.4737 | 0.000149 | 0.018077 | 1.28 |
| PPIAP80 | -0.73219 | 7.9174 | -5.4553 | 0.000153 | 0.018077 | 1.2519 |
| THUMPD1 | 0.62206 | 7.0126 | 5.4461 | 0.000155 | 0.018077 | 1.2379 |
| UBE2S | -0.85823 | 9.5663 | -5.4288 | 0.00016 | 0.018077 | 1.2113 |
| TTYH1 | 0.91975 | 6.5192 | 5.2119 | 0.000227 | 0.02107 | 0.87521 |
| PIM2 | 0.60154 | 11.337 | 5.1894 | 0.000235 | 0.02107 | 0.83983 |
| CAP1 | 0.60341 | 11.686 | 5.1859 | 0.000236 | 0.02107 | 0.83446 |
| UQCR11 | -0.76795 | 12.139 | -5.1783 | 0.000239 | 0.02107 | 0.8225 |
| PFN1P2 | -0.62436 | 12.287 | -5.1684 | 0.000243 | 0.02107 | 0.80682 |
| CEP170B | 0.76049 | 7.2506 | 5.1334 | 0.000258 | 0.021358 | 0.75167 |
| LDLR | 0.63924 | 8.3455 | 5.0897 | 0.000277 | 0.022337 | 0.68256 |
| TNFRSF1B | 0.66478 | 10.388 | 5.0386 | 0.000301 | 0.023263 | 0.60132 |
| PDCD1 | -0.6584 | 9.0109 | -4.9723 | 0.000336 | 0.024692 | 0.49532 |
| KLRC4 | -0.86794 | 7.8514 | -4.9683 | 0.000338 | 0.024692 | 0.48895 |
| OSTCP1 | -0.74731 | 7.912 | -4.9611 | 0.000343 | 0.024692 | 0.47745 |
| HTR1B | -0.69858 | 6.0376 | -4.9402 | 0.000355 | 0.025066 | 0.4439 |
| ARHGEF6 | 0.69158 | 7.3207 | 4.9178 | 0.000368 | 0.025524 | 0.40772 |
| ASB2 | -0.66516 | 8.5874 | -4.8972 | 0.000381 | 0.02592 | 0.37453 |
| NOMO1 | 0.59862 | 7.9424 | 4.8811 | 0.000392 | 0.025923 | 0.34855 |
| CCDC88A | -0.65359 | 7.1495 | -4.8708 | 0.000398 | 0.025923 | 0.33184 |
| ROMO1 | -0.58826 | 10.394 | -4.8678 | 0.0004 | 0.025923 | 0.32709 |
| POTEM | 0.65 | 13.44 | 4.7231 | 0.000511 | 0.028273 | 0.091352 |
| TMEM14C | -0.71074 | 9.8205 | -4.6837 | 0.000547 | 0.02913 | 0.026591 |
| ATF4 | -0.7045 | 13.843 | -4.5833 | 0.000649 | 0.031813 | -0.13925 |
| TOP1 | 0.76415 | 8.1293 | 4.5776 | 0.000655 | 0.031813 | -0.14881 |
| COX7C | -0.80146 | 11.19 | -4.5318 | 0.000709 | 0.03249 | -0.22485 |
| GPER1 | -0.98647 | 7.2134 | -4.5072 | 0.00074 | 0.033134 | -0.26589 |
| SRP68 | 0.59727 | 8.2496 | 4.4963 | 0.000754 | 0.033272 | -0.28403 |
| PACSIN2 | 0.7323 | 9.2176 | 4.4896 | 0.000763 | 0.033523 | -0.29523 |
| EEF1B2 | -0.81726 | 12.951 | -4.4725 | 0.000786 | 0.033767 | -0.32394 |
| NCOA1 | 0.75213 | 10.059 | 4.4715 | 0.000787 | 0.033767 | -0.32552 |
| VMA21 | -0.68969 | 7.7653 | -4.4387 | 0.000833 | 0.034412 | -0.38054 |
| USP5 | 0.59373 | 7.1139 | 4.4263 | 0.000851 | 0.03447 | -0.40135 |
| NDUFB1 | -0.66851 | 9.8297 | -4.4066 | 0.000881 | 0.035145 | -0.43449 |
| CD59 | 0.60472 | 5.7879 | 4.3931 | 0.000902 | 0.035714 | -0.45712 |
| AGAP3 | -0.62811 | 9.4757 | -4.3906 | 0.000906 | 0.035725 | -0.46143 |
| PRSS33 | -0.7195 | 6.4741 | -4.3844 | 0.000915 | 0.035725 | -0.4719 |
| COG7 | 0.58551 | 7.5788 | 4.3809 | 0.000921 | 0.035725 | -0.4777 |
| ZNFX1 | 0.64444 | 8.1478 | 4.3575 | 0.000959 | 0.035901 | -0.51728 |
| PHTF2 | -0.92939 | 7.7102 | -4.3001 | 0.001061 | 0.037852 | -0.61428 |
| H4C12 | -0.66552 | 7.146 | -4.2788 | 0.001101 | 0.038721 | -0.65041 |
| AMH | -0.67523 | 6.2269 | -4.2384 | 0.001182 | 0.040973 | -0.71918 |
| LSM6 | -0.66482 | 9.2167 | -4.2208 | 0.001219 | 0.041048 | -0.74911 |
| TMEM165 | 0.63976 | 7.2096 | 4.2006 | 0.001264 | 0.041531 | -0.78357 |
| INA | -0.66074 | 6.6036 | -4.1997 | 0.001266 | 0.041531 | -0.78511 |
| ZBTB32 | -0.72961 | 7.4726 | -4.1989 | 0.001267 | 0.041531 | -0.78651 |
| CLEC3B | -0.63736 | 8.8113 | -4.1609 | 0.001355 | 0.042855 | -0.85143 |
| COX6C | -0.75899 | 9.2459 | -4.1471 | 0.001389 | 0.043297 | -0.87494 |
| SELENOH | -0.74662 | 12.992 | -4.0919 | 0.001532 | 0.045308 | -0.96969 |
| ITGAV | -0.60343 | 6.8771 | -4.0655 | 0.001605 | 0.046541 | -1.0152 |
| TPT1-AS1 | -0.5933 | 9.3004 | -4.0465 | 0.001661 | 0.046779 | -1.0478 |
| SEC11C | -0.69401 | 9.8868 | -4.0405 | 0.001678 | 0.046779 | -1.0581 |
| GPR137B | -0.63724 | 7.2715 | -4.0177 | 0.001748 | 0.047094 | -1.0974 |
| CXCL5 | -0.61822 | 8.9867 | -4.0073 | 0.001781 | 0.047202 | -1.1155 |
| GGACT | -0.86078 | 7.5342 | -3.9977 | 0.001811 | 0.047661 | -1.132 |
| BICRA | -0.63886 | 7.0575 | -3.9628 | 0.001928 | 0.049225 | -1.1924 |
| TSPAN4 | -0.70821 | 8.648 | -3.9606 | 0.001936 | 0.049225 | -1.1962 |
| OR1L3 | 0.77108 | 5.884 | 3.9582 | 0.001944 | 0.049225 | -1.2004 |
| LYRM9 | -0.60185 | 9.3425 | -3.955 | 0.001955 | 0.049225 | -1.2059 |
| SSTR5-AS1 | -0.99916 | 7.3952 | -3.9541 | 0.001959 | 0.049225 | -1.2075 |
| LSM8 | -0.69742 | 8.7651 | -3.9442 | 0.001994 | 0.049334 | -1.2246 |

Supplementary Table 2 The top five targeted genes regulated by miRNA

| Gene | Degree | Betweenness | Expression |
| --- | --- | --- | --- |
| LDLR | 167 | 202450.28 | 0.639243 |
| POTEM | 124 | 124732.6 | 0.650004 |
| IFNAR2 | 109 | 113646.6 | 1.474765 |
| BAZ2A | 107 | 125410.61 | 0.703175 |
| SRF | 92 | 91198.44 | 0.646737 |

Supplementary Table 3 The top five targeted genes regulated by transcription factor

| Gene | Degree | Betweenness | Expression |
| --- | --- | --- | --- |
| SRF | 25 | 1021.09 | 0.6467371 |
| TSPAN4 | 18 | 604.48 | -0.708209 |
| CD59 | 16 | 472.8 | 0.6047165 |
| ETS1 | 16 | 336.97 | 1.1452451 |
| SLC25A25 | 15 | 354.57 | -0.618533 |
